# Qualitative Evidence Synthesis on Self-Collection for Human Papillomavirus–Based Cervical Screening: Protocol for Systematic Review

**DOI:** 10.2196/21093

**Published:** 2020-10-22

**Authors:** Hawa Camara, Ye Zhang, Lise Lafferty, Andrew Vallely, Rebecca Guy, Angela Kelly-Hanku

**Affiliations:** 1 The Kirby Institute UNSW Sydney Wallace Wurth Building Kensington Australia; 2 Centre for Social Research in Health UNSW Sydney Kensington Australia; 3 Papua New Guinea Institute of Medical Research Goroka, Eastern Highlands Province Papua New Guinea

**Keywords:** self-collection, HPV-based testing, cervical screening, qualitative evidence synthesis, protocol, systematic review

## Abstract

**Background:**

Cervical cancer is the fourth most common cancer affecting women worldwide. In the 1980s, it was found that the sexually transmitted disease human papillomavirus causes over 90% of all cervical cancer cases. Since that discovery, diagnostic technologies have been developed for the detection of human papillomavirus DNA in cervical samples. However, significant sociocultural and structural barriers remain. Considerable strides have taken place in recent years to address these barriers, such as the self-collection for human papillomavirus–based cervical screening method.

**Objective:**

The purpose of this review is to synthesize qualitative evidence around the self-collection method and identify strategies to increase acceptability and feasibility in different settings. This qualitative synthesis will be used to better understand how to conceptualize and implement more effective, accessible, and socially and culturally acceptable cervical screening programs and policies globally.

**Methods:**

A systematic search will be conducted in Global Health, Cochrane, CINAHL (Cumulative Index to Nursing and Allied Health Literature), ProQuest, ScienceDirect, EMBASE, EMCARE, Medline (OVID), Scopus, and Web of Science. Published and peer-reviewed articles will be included. Two reviewers will independently screen and assess the studies. The data will be coded and analyzed using a thematic synthesis process. The socioecological model will be used to organize emergent themes at the micro and macro levels. The results will be presented in narrative and tabular form.

**Results:**

The article search and data extraction were completed in May 2020. The data were analyzed in June 2020. The review will be submitted for publication in Fall 2020.

**Conclusions:**

This review will present the global evidence of the perspectives and experiences of various key stakeholders and how these perspectives and experiences impact their decision-making process to perform or accept self-collection for human papillomavirus–based cervical screening. The review will provide guidance to implementation researchers as well as implications for future research.

**Trial Registration:**

PROSPERO International Prospective Register of Systematic Reviews CRD42019109073; https://www.crd.york.ac.uk/PROSPERO/display_record.php?RecordID=109073

**International Registered Report Identifier (IRRID):**

DERR1-10.2196/21093

## Introduction

Cervical cancer is the fourth leading cause of cancer affecting women in the world, with the highest burden of disease, estimated at 85% [1], occurring in low- and middle-income countries. To attenuate the global cervical cancer burden, the implementation of screening methods is a global health priority. The most commonly known screening method is cytologic screening with a Papanicolaou test (Pap smear): since its introduction in the 1940s, cervical cancer incidence and mortality have decreased by 70% in high-income countries [2]. However, Pap smears require distinctive and extensive laboratory infrastructure and capacity, making it challenging to implement in resource-limited settings [3,4].

In the early 1980s, researchers discovered the presence of human papillomavirus (HPV) DNA in cervical cancer lesions and that the sexually transmitted infection (STI) was responsible for over 90% of malignant cases [5]. Subsequent research has demonstrated that persistent HPV infection puts women at higher risk for cervical cancer [6]. These research findings have led to the development of tests for the detection of oncogenic HPV types in cervical precancerous or cancerous lesions, referred to as HPV testing. Additionally, this was an opportunity to develop technologies that are low-cost and require minimal laboratory infrastructure and training to use. Since this discovery of HPV oncogenic types, technologies for conducting HPV testing have revolutionized the way the global health community views cervical screening and its impact on global health outcomes [7]. The new screening methods offer access to screening services at a relatively low-cost via an effective and efficient process that bypasses all laboratory needs required by the Pap smear. Most of the research to date has been focused on determining the effectiveness of HPV-based cervical screening methods. A global review by Kouliopoulos et al [8] of studies conducted between 1992 and 2015 showed that HPV testing has higher sensitivity and specificity than those of conventional cytology methods and allows for less frequent screening. When provided at point-of-care, patients receive their results in a shorter time frame (60-90 minutes on average, compared to up to 2 weeks for Pap smears) allowing for faster turnaround, more timely treatment, and reduction in patients lost to follow-up [4,9,10]. Research from Goldie et al [11] demonstrated that HPV-based screening for women once they have reached 35 years of age potentially reduces the lifetime risk of cervical cancer by 36%.

The concept of self-collection (interchangeably named self-collected samples or self-sampling) was introduced in the 1970s to address the alarmingly low rates among women who lacked access to screening and health care services, often due to sociocultural, economic, and structural factors [12]. In the 1990s, Dr. Arthur Fournier noticed the concerning underutilization rates of Pap smear screening in Haiti. To circumvent the discomfort and embarrassment of pelvic examinations, a known deterrent for women seeking cervical screening services, Dr. Fournier designed a cervical self-sampling device for the detection of cancer and STIs [13]. The innovative self-sampling device that could also be used at home provided an efficient solution to screen women in resource-limited and culturally challenging settings. Since then, numerous studies [14-17] have compared the efficacy and effectiveness of self-collected samples to clinician-collected samples for cervical screening. The evidence showed that self-collected sampling not only had comparable sensitivity and specificity to those of clinician-collected sampling but was also the most-widely preferred screening method by women. Other research [18] has demonstrated that self-collection of specimens for HPV testing could potentially increase uptake for women residing in hard-to-reach settings.

With the vast and rapid progress in cervical screening methods, it is essential to recognize that, when applied in varying contexts and among different people, responses and experiences will significantly differ. In the case of self-collection for HPV screening, social values, cultural values, and understanding of the female body can each have an impact on women's acceptability of innovative cervical screening methods. To be successful, methods such as self-collection, need to be socially and culturally accepted by women, health care workers, and policymakers [19], as well as fit within settings where access to screening is scarce due to sociocultural barriers and limited resources. This requires understanding the experiences of all key actors (ie, women, health care workers, and policymakers) at all levels of the health system interacting with it.

This review aims to generate findings for further guidance to implementation researchers. It will help with the design of self-collection for HPV-based cervical screening interventions that address all factors raised by key stakeholders that could impact the acceptability and feasibility of these programs in diverse cultural and geographical settings.

To facilitate the uptake of cervical screening, thus reducing the incidence and prevalence of cervical cancer globally, this systematic review aims to identify, analyze, and synthesize the experiences and perceptions of women, health care workers, and policymakers. Consequently, it will provide evidence of factors that impact the acceptability and feasibility of self-collection for HPV-based cervical screening globally.

## Methods

### General

A preliminary search for existing systematic reviews on the topic has been conducted in major databases (ie, Medline; Scopus; Joanna Briggs Institute Database of Systematic Reviews and Implementation Reports; Cochrane Database; CINAHL, Cumulative Index to Nursing and Allied Health Literature; PubMed; and PROSPERO, International Prospective Register of Systematic Reviews). We found no qualitative synthesis systematic reviews that explored factors that could impact the acceptability and feasibility of self-collection for HPV-based cervical screening from the perspectives of women, health care workers, and policymakers, globally. The protocol will follow PRISMA (Preferred Reporting Items for Systematic Reviews and Meta-Analyses) guidelines [20]. Participant, interest, and context inclusion criteria [21] used for this review are detailed.

### Review Questions

What are women’s, health care workers’, and policymakers’ experiences with and perceptions of self-collection for HPV-DNA cervical cancer screening?

What are the barriers and facilitators to the acceptability and implementation of self-collection for HPV-DNA cervical cancer screening from the point of view of women, health care workers, and policymakers?

### Types of Participants

The review will focus on women, health care workers, and policymakers. For women participants, there will be no age restriction since the review will focus on both perspectives and experiences of the self-collection method, albeit the HPV-DNA test is recommended for women 30 years old and above only. Also, the reviewers will allow for different terms to be used when identifying the types of participants targeted in this review: for example, health care workers could be defined differently in studies (ie, providers, health care service providers, etc).

### Phenomena of Interest

The intervention of interest in this review is self-collection for HPV-based cervical screening. This review will consider all elements associated with the process of self-collection. The first outcome will be to assess the sociocultural and structural barriers and facilitators from the point of view of women (ie, patients), health care workers, and policymakers as well as to determine the feasibility factors of self-collection for HPV-based cervical screening in different settings.

### Context

The review will not have a global geographical restriction: all countries with publicly available data will be included. Additionally, any setting where self-collection for HPV-based cervical screening could be performed (which includes women’s homes, primary care, community health center) will also be identified and included in the review.

### Study Search Methods

#### Types of Studies

This review will consider qualitative and mixed methods studies (Textbox 1) that draw on the experiences and perspectives of women, health care workers, and policymakers. These will include designs such as grounded theory, narratives, ethnography, phenomenology, and action research.

Two authors will independently screen the database search results, compare and discuss their findings, and resolve disagreements to reach consensus.

**Criteria.**

**Inclusion criteria**

- Qualitative studies (qualitative component of mixed methods studies, interviews, focus groups, surveys, or questionnaires with open-ended questions) that explored experiences, perspectives of self-sampling or self-collection for human papillomavirus (HPV)–based cervical screening
- Studies that involve women; health care workers including physicians (obstetrics/gynecology primarily), nurses, midwives, and allied health professionals; or policymakers
- Studies published in English
- Studies in any geographical setting (high-income countries and low- and middle-income countries) and health care settings (eg, community health care centers, primary health care centers, patient's homes)
- Peer-reviewed publications published after 1986 (year of the first study on HPV-DNA testing)

**Exclusion criteria**

- Studies that involve stakeholders other than the ones listed in the inclusion criteria
- Studies in languages other than English
- Purely quantitative studies and quantitative components of mixed methods studies
- Nonpeer reviewed articles, theses, abstracts, reviews, or book chapters
- Any other cervical screening method that is not self-collection for HPV-based cervical screening (ie, clinician-collected cervical screening, Pap smears, visual inspection with acetic acid or visual inspection with Lugol iodine)

#### Search Strategy

A systematic search of the following databases will be developed in collaboration with a librarian and conducted by HC in Global Health, Cochrane, CINAHL (Cumulative Index to Nursing and Allied Health Literature), ProQuest, ScienceDirect, EMBASE, EMCARE, Medline (OVID), Scopus, and Web of Science. Published peer-reviewed articles will be included.

The literature search will be limited to studies published in English, between 1986 (year of the first study on HPV-DNA testing) to December 2019. It will ensure that both controlled vocabulary, medical subject headings (MeSH) and keywords, are tailored to each database (see an example of the draft EMBASE search strategy and keywords used in Multimedia Appendix 1.

The search strategy will include terms focused on 4 main concepts: HPV, self-collection, HPV-DNA testing, and qualitative. The primary reviewer HC will consult with the librarian for help refining the search strategy and terms to ensure the inclusion of both Medical Subject Heading (MeSH) terms and keywords relevant to this review and its aims. A preliminary list of MeSH terms and keywords are listed in Table 1.

**Table 1:** Terms and keywords.

Concept	Keywords	MeSH^a^
HPV^b^	*Human papillomavirus* OR *human papilloma virus*	*Papillomaviridae* OR *papillomavirus infections* OR *uterine cervical neoplasms*
	AND	AND
HPV-DNA testing	*Cervical cancer screening* OR *cervical screening* OR *cervical ADJ8 screening*	*HPV-DNA testing* OR *HPV testing* OR *HPV* primary testing OR *primary HPV testing* OR *DNA probes, HPV* OR *human papillomavirus DNA tests* OR *vaginal smears*
	AND	AND
Self-collected	*Self-sampling* OR *self-collected* OR *self-administered*	—^c^
	AND	AND
Qualitative	*Qualitative*	*Qualitative research* OR *qualitative studies* OR *qualitative study* OR *focus groups* OR *interviews as topic* OR *observation* OR *ethnography*

^a^MeSH: Medical Subject Headings.

^b^HPV: human papillomavirus.

^c^No MeSH terms were used.

All articles that are identified will be imported into EndNote (QSR International) to systematically sort, review, and select the final list of articles to be included in the synthesis. Guided by the eligibility criteria, 2 reviewers (HC and YZ) will screen each article in the Endnote library by title and abstract. The list of articles subjected to full review will be agreed upon between the 2 reviewers (HC and YZ). The 2 reviewers will proceed to review the full text of all eligible studies. The final list of included studies will be discussed and agreed upon between the 2 reviewers and an additional third reviewer (AKH).

### Assessment of Methodological Quality of Included Studies

All included studies will be critically appraised using the Critical Appraisal Skills Programme tool or CASP for qualitative research [22], albeit there is no clear consensus on a standard tool applied to the methodological appraisal of qualitative studies [23]. This will be conducted independently by 2 reviewers (HC and YZ). The CASP tool consists of 10 questions that assess the essential elements of qualitative research. Each item will be scored using yes (1 point), no (0 points), or “can’t tell” (0.5 points) to score the article's quality out of 10.

The quality of the article will be determined using the following scoring guideline: a score ≤3 is of low quality, a score ranging from 4-6 is of medium quality, and a score ≥7 is of high quality. The appraisal score will not be used to exclude articles.

### Data Extraction And Analysis

#### Data Extraction

As an initial step, an extraction exercise will be conducted to ensure accuracy, completeness, and richness of the data. An Excel (Microsoft Inc) data extraction spreadsheet will be developed to include the following study characteristics: title, authors, journal, publication date, study design, research aim, setting and study location, sample size, age group, demographics, intervention or screening type, data collection method, theoretical or conceptual framework, data analysis, outcomes, and findings.

Two reviewers (HC and YZ) will independently extract the data using the data extraction spreadsheet, and any disagreements will be discussed to reach consensus.

#### Data Synthesis

Qualitative research findings will be pooled using the thematic synthesis approach. This approach, developed by Thomas and Harden [24], specifically looks at individuals’ perspectives and experiences [25] using an integrative approach which considers data from comparable primary studies. For data coding, we will use NVivo (version 12.5; QSR International) qualitative data management and analysis software. Codes will inductively be identified and grouped into themes [26]. The thematic synthesis approach is comprised of 3 stages: coding text where each study will be coded line-by-line extracting data that responds to the research questions developed for this review (HC will conduct this); developing descriptive themes where the codes identified in the first stage will be categorized based on similarities to create themes (HC will conduct this); and generating analytical themes where the themes identified in the second stage will be used to develop key messages (this will be conducted by HC and discussed with and agreed upon by YZ, LL, AV, RG, and AKH).

As part of the thematic synthesis, a theoretical framework was identified to guide the data synthesis process. For this review, the socioecological model will be the a priori framework that will need to be discussed and approved by all reviewers (HC, YZ, LL, AV, RG, and AKH), used, and adapted to identify emerging themes. Socioecological model views health behavior as being shaped by and influenced at multiple levels [27]: (1) intrapersonal, (2) interpersonal, (3) organizational, (4) community, and (5) public policy. By using the socioecological model, we aim to identify micro and macro factors that impact the perspectives and experiences of using self-collection for HPV testing, thus impacting acceptability from the point of view of all key stakeholders, as well as the implementation of the self-collection method in different settings.

The findings will provide a descriptive summary of key themes and associated quotations.

### Patient and Public Involvement

This review will include solely secondary data, patients, health care workers, policymakers, and the public will not be involved in the design or conduct of the review.

### Ethics and Dissemination

This review will involve the collection and analysis of publicly available secondary data, and therefore, does not require ethical approval. The review findings will be disseminated through publication in a peer-reviewed journal and scientific conference presentations. The review will include relevant discussion points to further guide implementation researchers for the prevention of cervical cancer globally as well as implications for future research.

## Results

The protocol was registered on April 15, 2019 (PROSPERO CRD42019109073). The article search and data extraction were completed in May 2020. The review includes 33 papers published between 2008 and 2020. The data were analyzed in June. The review will be submitted for publication in Fall 2020.

## Discussion

Qualitative research should be a priority very early on in implementation research when introducing socially and structurally sensitive screening programs. This qualitative evidence synthesis will aim to review the perspectives and experiences of key stakeholders and the impact on their decision-making process to perform or accept self-collected HPV-based cervical screening. To date, scoping and systematic reviews on cervical screening have focused on qualitative evidence about Pap smears and visual inspection with acetic acid methods, and most recently, HPV testing. This will be the first review that qualitatively explores the most recent innovative HPV-testing method of self-collection, which is being increasingly used globally. By using the socioecological model, strategies will be identified and discussed to address barriers and facilitators to increase acceptability and feasibility at every level of the system. The review will include relevant discussion points to further guide implementation researchers for the prevention of cervical cancer globally as well as implications for future research.

## Appendix

Multimedia Appendix 1 EMBASE draft strategy.

## Abbreviations

CASP: Critical Appraisal Skills Programme
CINAHL: Cumulative Index to Nursing and Allied Health Literature
DNA: deoxyribonucleic acid
HPV: human papillomavirus
MeSH: Medical Subject Headings
PRISMA: Preferred Reporting Items for Systematic Reviews and Meta-Analyses
STI: sexually transmitted infection
