# Supplementary material for: Qualitative Evidence Synthesis on Self-Collection for Human Papillomavirus–Based Cervical Screening: Protocol for Systematic Review
Source: JMIR Res Protoc. 2020 Oct 22;9(10):e21093. doi: 10.2196/21093 (PMC7644378; doi:10.2196/21093)
Supplement: Multimedia Appendix 1 [file resprot_v9i10e21093_app1.docx]

| 1. wart virus/ or papillomaviridae/ |
| --- |
| 2. human papillomavirus.mp. |
| 3. HPV.mp. |
| 4. 1 or 2 or 3 |
| 5. (cervical adj4 screening).mp. [mp=title, abstract, heading word, drug trade name, original title, device manufacturer, drug manufacturer, device trade name, keyword, floating subheading word, candidate term word] |
| 6. uterine cervix cancer/ |
| 7. (HPV adj4 testing).mp. [mp=title, abstract, heading word, drug trade name, original title, device manufacturer, drug manufacturer, device trade name, keyword, floating subheading word, candidate term word] |
| 8. qualitative research/ |
| 9. qualitative analysis/ |
| 10. qualitative.mp. |
| 11. focus group.mp. |
| 12. interview/ |
| 13. 5 or 6 or 7 |
| 14. 8 or 9 or 10 or 11 or 12 |
| 15. 4 and 13 and 14 |
| 16. Human papillomavirus DNA test/ or HPV-DNA testing.mp. |
| 17. 13 or 16 |
| 18. 4 and 14 and 17 |
| 19. self-sampling.mp. |
| 20. self-collected.mp. |
| 21. 19 or 20 |
| 22. 18 and 21 |
| 23. interview/ or semi-structured interview/ |
| 24. in-depth interview.mp. |
| 25. ethnography/ |
| 26. 8 or 9 or 10 or 11 or 23 or 24 or 25 |
| 27. HPV based.mp. |
| 28. 17 or 27 |
| 29. 4 and 26 and 28 |
